# A Comparison of the Effectiveness of Online Instructional Strategies Optimized With Smart Interactive Tools Versus Traditional Teaching for Postgraduate Students

**DOI:** 10.3389/fpsyg.2021.747719

**Published:** 2021-12-23

**Authors:** Ping Wang, Teng Ma, Li-Bo Liu, Chao Shang, Ping An, Yi-Xue Xue

**Affiliations:** Department of Neurobiology, School of Life Sciences, China Medical University, Shenyang, China

**Keywords:** online, face-to-face, smart interactive tools, effectiveness, postgraduate course, neurobiology, instructional strategies, interaction

## Abstract

To solve the problem that lack of interaction in online courses affects motivation and effectiveness of students’ learning, smart interactive tools were introduced into the online Neurobiology course. This study aimed to evaluate the students’ satisfaction with online teaching mode and assess the academically higher and lower performing students’ learning effectiveness in the online course optimized with smart interactive tools compared to face-to-face learning. Descriptive statistics and independent *t*-tests were used to describe student samples and determine the differences in students’ satisfaction and performance. Reflections of students’ satisfaction revealed that about 65.8% were satisfied with the learning involvement and about 60.5% were satisfied with the class interaction. Almost two-thirds of the class agreed that the smart interactive tools applied in the online course could help them attain their learning goals better. Among all the smart interactive functions, the class quiz was the most effective one in helping students grasp the main points of the course. No significant differences were found between the two teaching modes in the overall and academically higher or lower performing students’ final exam average scores. Compared to each band score of such two teaching modes, no one failed to pass the final exam in the online course, however, three lower-performing students who were taught in the traditional course failed. This study suggested that optimized online teaching with smart interactive tools could produce the same learning effectiveness for the academically lower-performing students as for the higher-performing students. Meanwhile, the instructors could know the learning status in which each student was and perform personalized guidance and improve exam passing rate accordingly.

## Introduction

With the rapid progress of the integration of education and information technology in the digital era, the online teaching mode and teaching strategies of postgraduate courses need to be optimized. Compared to the traditional or face-to-face teaching mode, the previous online teaching mode has several problems as follows: (1) Due to the lack of teacher’s supervision, it is impossible to ensure that the students would always stay focused in class. (2) Since teacher and students are not in the same space, it is difficult to make real-time interaction. The teacher cannot know whether or not the students understand the teaching content through their facial expressions and feedback. (3) Online teaching has a different but professional requirement for the teaching infrastructure or ecosystem. At present, the application of online teaching tools and MOOC resources has greatly promoted the development of online teaching modes in higher education (Curran et al., 2019; Pozón-López et al., 2021). Much of the research had indicated that compared to face-to-face instructional delivery format, the student evaluation of online courses showed no significant differences both inside and outside the health education field (Riffell and Sibley, 2005; Campbell et al., 2008; Todd et al., 2017; Harwood et al., 2018). For some basic professional courses in science and engineering, which need more hands-on activities and live demonstrations, online teaching still has some issues on students’ motivation and effectiveness (Akdemir, 2010). The questionnaire from Tang et al. (2020) showed the undergraduate engineering students were dissatisfied with the communication and Q&A modes in online classes. Moreover, some courses, which required higher cognitive skills of analyzing, evaluating, and creating, such as statistics, might produce poorer test performance to teach online among the students with lower cognitive skills of remembering and understanding (Lu and Lemonde, 2013). For the academically lower-performing students, the lack of face-to-face synchronous interaction of online courses might be the reason for their worse test performance compared to the academically higher-performing students. In addition, studies have shown that online courses that lack substantive and meaningful interaction might generate a sense of isolation, unsatisfying learning experiences, and high dropout rates (Bennett et al., 1999). Therefore, how to solve the lack of interaction in online courses is essential for improving students’ motivation and effectiveness in learning.

Recently, technical requirements (e.g., Blackboard, MOODLE, Web 3.0, email, discussion boards, and Internet speed) and learning skills (e.g., motivation, social interaction, and self-discipline) were introduced into online courses to solve the above problems (Cho et al., 2010; Cho, 2012; Aljawarneh, 2020; Müller and Wulf, 2020). Furthermore, some smart interactive tools, such as Rain Classroom and Tencent Meeting, are another technical choice to improve this problem (Lu and Ding, 2020; Zhang, 2020). At present, Rain Classroom has been successfully used for online teaching of different subjects, such as biochemistry and English (Shu et al., 2019; Wu, 2019; Wang and Hu, 2020) and realizes the interaction between the learners and the content, as well as the learners and the instructor. This smart interactive tool not only has various interactive functions, but also has a data analysis function. The data analysis function could help the instructor to grasp the students’ learning situation of the course content and to optimize the teaching strategy conveniently through the feedback in time from students’ preview and review performance, the quiz answers in and after class, and the final test scores as well. Tencent Meeting is also a widely used interactive tool, which can mainly realize the learner-instructor interaction as well as the inter-learner interaction. The introduction of these smart interactive tools solves the lack of synchronous interaction and feedback, improves the student learning motivation, and makes online teaching in higher education more effective.

Allen and Seaman (2015) pointed out many higher education institutions accepted that online learning is critical to their long-term strategy. Campbell et al. (2008) in their study showed that compared to undergraduate level, graduate students who have higher scholastic aptitude could produce better test performance with an online or hybrid online course. In the postgraduate courses, the online teaching mode will bring the following advantages: (1) Not restricted by the classroom resources and the number of students, and the teaching time can be arranged more flexibly (Peacock et al., 2012). (2) The students could learn using a self-paced and student-centered approach and review the course using the playback function (Ituma, 2011; Kemp and Grieve, 2014). The graduate students studying online are often required to take on greater responsibility for their own learning. Compared to face-to-face learning, students in the online course report feelings of social disconnectedness, missing familiar teacher immediacy, and interpersonal interactions and social cues (Slagter van Tron and Bishop, 2009; Crow and Murray, 2020). Therefore, introducing smart interactive tools into the online teaching of postgraduate courses is worth exploring and practicing. Lamon et al. (2020) proposed that a more active and interactive mode of online teaching provides postgraduate students with a greater sense of inclusion and satisfaction.

During the COVID-19 pandemic in the 2020 spring semester, the adoption of online learning seems to be an abrupt response to the crisis (Bozkurt and Sharma, 2020). Charles et al. (2020) defined this temporary shift of instructional delivery to an alternate delivery mode without the building of a specially designed ecosystem–emergency remote teaching (ERT). The Neurobiology elective course arranged to teach online this spring semester doesn’t belong to ERT. Before online teaching, all teachers have received technical training on smart interactive tools since 2019 and designed the online instructional strategies as well. In addition, the instructors also have 1 year experience in blended teaching mode for undergraduates. York et al. (2007) pointed out that many online courses are not designed or delivered with careful consideration of foundational instructional design principles. To improve the interaction and sense of presence, we optimized the instructional strategies, such as reducing the teaching time of one section and distributing some easy course content to preview materials to save more time for interaction with the students. Whether the new smart interactive tools and the optimized instructional strategies might improve the students’ effectiveness need to be explored.

To solve the problem that the lack of interaction of face-to-face courses in online courses affects the students’ motivation and effectiveness in learning, and then affects student test performance, smart interactive tools were introduced into neurobiology online courses. The aim of this study is to assess the students’ satisfaction of the attitude and practice toward the Neurobiology online course optimized with the smart interactive tools and to evaluate the academically higher and lower performing students’ learning effectiveness of the two teaching modes. We hope to solve the lack of interaction in online teaching, find out the satisfying online instructional strategies and course design features, and enhance students’ learning effectiveness with smart interactive tools.

## Materials and Methods

### Study Participants

This study assessed 74 neurobiology students’ reflections about the Neurobiology course. Of whom, 38 students were enrolled in the online course, their average age was 27 ± 2.5 years, with ages ranging from 23 to 35 years. Forty-seven percent of students were female. 36 students were enrolled in the traditional course, their average age was 26 ± 2.3 years, with ages ranging from 24 to 31 years. Thirty-three percent of students were female.

### Study Design

This study was intended to examine the effectiveness of the online course design features and instructional strategies optimized with the smart interactive tools from the students’ perspective. The teachers of online courses are all professors and associate professors, who have been teaching the traditional face-to-face Neurobiology course for years. They designed the instructional strategies of this online course. The total teaching time of the two teaching modes is the same. The subjects are the postgraduate students who choose this Neurobiology course.

### Context of the Study

The neurobiology course is an elective course offering for all postgraduate students. The theoretical part of this course has been taught offline in the past, with a teaching time of 3 weeks, 12 sections. To cultivate physicians and scientific researchers with a broad neuroscience foundation and solid scientific research skills, the learning objectives of this course are as follows:

• Knowledge goal: To expand and deepen the theoretical knowledge of neurobiology, especially the advanced brain functions, neuroendocrine-immune regulatory network, and related disciplines such as neurology and psychiatry, to know the frontiers of neurobiological development.
• Ability goal: To master the method to establish common central nervous system disease models, to independently conduct scientific research in neurobiology and related disciplines, to promote the translational research of neuroscience.
• Literacy goal: To perform a rigorous scientific attitude, to stick to academic ethics.

The course content includes the molecular mechanisms and research models of the latest advances in learning and memory, Alzheimer’s disease, Parkinson’s disease, epilepsy, alcohol-related neurologic disorders, and other central nervous system diseases. At the same time, relevant experimental courses were set up for the establishment and evaluation of several brain-disease models, such as the glioma model, cerebral ischemia model, and depression model, etc. So far, the content of experimental courses is not suitable for online teaching. The online teachers, teaching time, teaching purpose, teaching content, and final evaluation of this study are completely consistent with those of traditional teaching.

### Online Smart Interactive Tools

**FIGURE 1:**
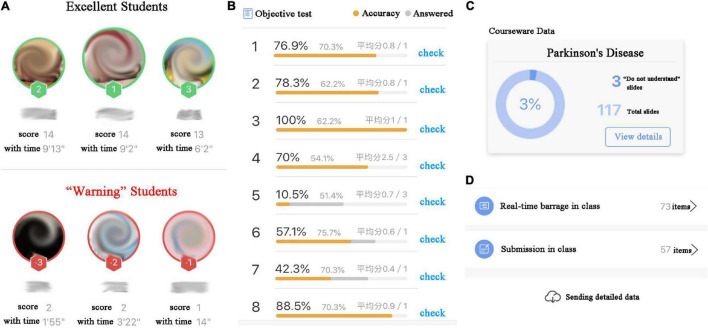
Part functions of smart interactive tool Rain Classroom. **(A)** Participation in the class quiz. **(B)** Correct rate of quiz answers. **(C)** The courseware data of the students who did not understand a certain slide of PowerPoint. **(D)** The data of the students who posted the real-time barrages, who sent the submission in online class.

In online teaching, the appropriate smart interactive tools are very crucial to achieve satisfactory teaching effectiveness. Based on the teaching experience of other online courses and the survey of students, the cloud-based Rain Classroom and Tencent Meeting tools were selected to jointly build the online teaching platform of the Neurobiology course. As a plug-in of PowerPoint, Rain Classroom can enable PowerPoint combined with audio/video live broadcast for thousands of people, can also be used to send preview and review materials before and after class, send quizzes, vote in class, and realize real-time interaction with students *via* the functions of the real-time barrage, submission, random roll call, and class bonus. Teachers can also check the students’ class participation using the functions of quiz answer time and accuracy rate, partial ranking of outstanding students, and early warning students (Figure 1). To realize the online face-to-face interaction through audio and video between teachers and students, Rain Classroom needs to be nested within Tencent Meeting, a cloud-based video conferencing tool. In addition, a WeChat group including the online teachers and students is established. If the students have any questions about any point in the course, they can communicate with the teachers through WeChat privately after class. The Rain Classroom also provides a course playback function for the students who are unable to log into the online class.

### Course Setup

This course was directly binding to the online teachers’ personal system of the smart interactive tools by the office of academic affairs. The online teachers built the resource bank and test bank of neurobiology in Rain Classroom, including courseware, preview and review PowerPoint, MOOC, and literature materials, self-made videos, and quizzes, etc. In the offline courses of Neurobiology, each section is approximately 45 min long, with a break of 5 min between two sections. Based on the teaching experience of other online courses and the survey of students, the online teaching time of each section is set up to 30 min, with a break of 5 min to ensure the online learning situation of students. The lack of appropriate and deep interaction between students and teachers is a common issue in online teaching, which may result in a sense of isolation and a high dropout rate (Moore, 1991; Ruth et al., 2015). To increase the interaction between students and teachers in a 30-min section, Rain Classroom provides several functions, such as timed quizzes, voting, real-time barrage, submission of comments, random roll call, and the detailed analyzed data. These functions would be helpful to ensure the students’ preoccupation (Figure 1).

### Assessment

To evaluate whether the smart interactive tools might improve the learning effectiveness of the academically lower-performing students, according to Garavilia and Gredler (2002) and Lu and Lemonde (2013) method, we subdivided the students in each teaching delivery group into academically higher and lower performing students using their assignment marks. The same assignments were given to both online and traditional face-to-face students, which are composed of multiple-choice questions and short answer questions. The assignments were given out once a week for 3 weeks during the course. After the subdivision, we had 18 lower-performing students, and 20 higher-performing students in the online class. In the face-to-face class, there were 17 lower-performing students and 19 higher-performing students. The median assignment mark for each student was computed. Online student assignment median was 85, while face-to-face students had an assignment median of 83.

To improve students’ enthusiasm for online learning, the test scores of this course are divided into two parts: formative score accounted for 10% of the total score (including the overall performance of student’s preview before class and review after class, class participation, and quiz scores), report writing score accounted for 20% of the total score, and 70% of the final exam score. When comparing with traditional courses, this study only compares the final exam scores due to the different assessment strategies of offline and online courses on the formative score. The final exam paper can be posted online through the Rain Classroom test bank. Rain Classroom also has a function of online invigilation, which is to identify students and supervise the students’ answering process through the computer camera.

### Data Analysis

Likert scale rating questions were used to assess students’ satisfaction with online courses. Students’ satisfaction with the online course of Neurobiology was displayed as proportions. Results were saved and analyzed using GraphPad Prism 7. Descriptive statistics were displayed in percentages and means ± standard error (SE). Comparison of online versus traditional assignment scores and the effectiveness of two teaching modes of Neurobiology course regarding students’ course evaluation scores was done using an independent *t*-test and a *P* value of ≤0.05 was considered significant.

## Results

### Analysis of the Students’ Performance Between Assignments and Final Exam

**FIGURE 2:**
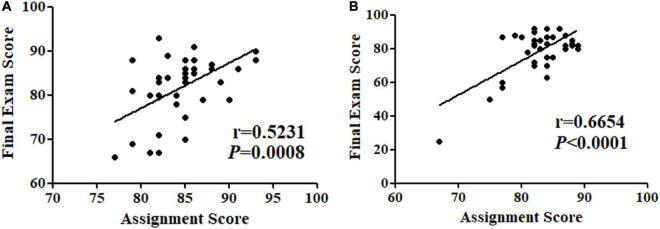
Correlation of final exam score with assignment score for online **(A)** and traditional face-to-face students **(B)**.

The correlation between the assignments and final exam performance from both online and traditional face-to-face students was analyzed to ensure the student’s assignment score could be used as a categorization method to classify a student’s scholastic aptitude. There was a significant correlation of 0.52 and 0.67 between the assignment average score and final exam performance (Figure 2). Then, the assignment performance was evaluated to determine if the students of the two-teaching mode had any significant differences in their aptitude for the Neurobiology course. No significant difference was observed in the overall average scores, as well as in the average scores of the lower and higher-performing students between the two teaching modes (Table 1).

**TABLE 1:** Comparison of online versus traditional assignment scores.

	Above median	Below median	Overall
	Mean (SE)	Mean (SE)	Mean (SE)
Online	87.20 (0.60)	81.17 (0.50)	84.34 (0.63)
Traditional	85.74 (0.47)	79.47 (0.97)	82.78 (0.74)
*P*-value	0.06	0.13	0.11
t-score	1.91	1.56	1.62
df	37	24	72

### Analysis of the Students’ Reflections

**FIGURE 3:**
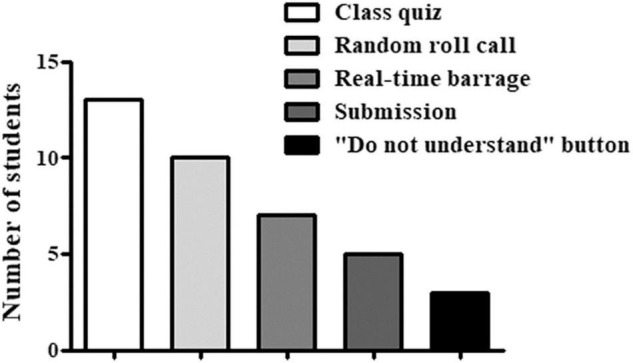
The students’ assessment of online instructional strategies.

Reflections of students about online Neurobiology courses using the Likert scale rating (Table 2) showed that about 65.8% were satisfied with the learning involvement, and about 60.5% were satisfied with the class interaction. Almost two-thirds of the class (65.8%) agreed that the online course helps them attain the learning goals; 5.3% disagreed and 23.7% could not decide. According to the student reflections, all online interactive functions are helpful. Among them, the class quiz is the most effective one in helping students grasp the main points of the Neurobiology course (Figure 3).

**TABLE 2:** Assessment of students’ satisfaction about the online neurobiology course.

	Strongly disagree %	Disagree %	Neutral %	Agree %	Strongly agree %
Learning motivation	2.6	7.9	18.4	55.3	15.8
Learning involvement	5.3	5.3	23.7	52.6	13.2
Class interaction	5.3	10.5	21.1	50.0	10.5
Understanding of the concepts	7.9	10.5	21.1	50.0	10.5
Completing of the learning tasks	5.3	7.9	23.7	52.6	10.5
Reaching the learning goals	5.3	5.3	23.7	55.3	10.5

### Analysis of the Students’ Final Exam Performance

Comparison of the overall and academically lower and higher performing students’ final exam average scores of the two teaching modes was done using an independent *t*-test (Table 3). Results indicated no significant differences were found between them. In the online group, no one failed to pass the final exam. The student number and average score of the band score from 60 to 69 were higher than those in the traditional group, and these students were all academically lower-performing students. No big differences were observed on the band score above 70 between the two teaching modes. However, in the traditional group, three lower-performing students failed to pass the final exam (Table 4).

**TABLE 3:** Comparison of students’ final exam average score of the two teaching modes.

	Above median	Below median	Overall
	Mean (SE)	Mean (SE)	Mean (SE)
Online	84.50 (1.18)	78.11 (2.00)	81.47 (1.24)
Traditional	81.63 (1.64)	74.82 (4.30)	78.38 (2.24)
*P*-value	0.16	0.50	0.23
t-score	1.43	0.69	1.21
df	37	22	54

**TABLE 4:** Comparison of student number and average score on different final exam band score of the two teaching modes.

Score	Course structure	Mean	N (overall)	N (above median)	% of N (above median/overall)
≥90	Online	91.25	4	3	75
	Traditional	91.00	4	2	50
80–89	Online	84.52	23	11	48
	Traditional	84.00	21	13	62
70–79	Online	75.33	6	4	67
	Traditional	73.00	6	3	50
60–69	Online	67.00	5	0	0
	Traditional	61.50	2	1	50
≤60	Online	–	–	–	–
	Traditional	44.00	3	0	0

## Discussion

Online teaching mode has many advantages as we mentioned at the beginning of this article. To better improve online teaching, we optimized it with the smart interactive tools in the postgraduate Neurobiology courses in 2020. To avoid the above-mentioned problems of previous online teaching, we designed a new online instructional strategy combined with our teaching experience from other online courses. To reduce the inability of students to stay focused for a longer time in the online course due to the lack of teacher’s supervision, we shortened the teaching time of each section from 45-min offline to 30-min online. During the 30-min period, a variety of interactive functions of smart interactive tools were used, such as class quiz, random roll call, real-time barrage, submission, voting, and “do not understand” button to allow students to actively participate in class. The above functions have all been praised by the students, they agreed these functions would help them better focus on the teaching content.

Lu and Lemonde (2013) in their study showed that students who struggle academically might produce poorer test performance in the online course for the lack of interaction as in traditional courses. The students of higher scholastic aptitude, such as graduate students, had better performance in the online course (Campbell et al., 2008). Although the Neurobiology course offering for postgraduate might require less cognitive skills than some other courses, such as statistics, we sub-divided the students of the two teaching modes into academically higher and lower performing groups using their assignment median scores to compare to the students’ learning effectiveness. The data analysis function of the smart interactive tool Rain Classroom is of great help for teachers to know the learning status in which each student is and provide the personalized guidance. This is an advantage that the traditional teaching mode does not have. Similar to Lu’s results, the academically lower (or higher) performing students in the online course did not show any difference in their scholastic aptitude compared to those in the traditional course. By comparing the final exam average scores of overall and of the academically lower and higher-performing students between the online and traditional groups, no significant differences were observed. In contrast, Lu’s results indicated that the lower performing students showed a significantly poorer test performance in the online teaching mode. Our equal online learning effectiveness for academically lower and higher-performing students might be the result of the optimized online teaching mode with smart interactive tools, which solved the problems available with the previous online teaching mode about the lack of synchronous interaction in class.

The smart interactive tools used in our online class have various functions. Class quiz designed from the main points of each section is the most popular one among the interactive functions. Random roll call makes students not dare to distract in class in case they do not know how to answer the questions. Real-time barrage allows all students to answer the question together. The “do not understand” button is suitable for shy students who do not like to communicate with the teacher through real-time interaction. By clicking the “do not understand” button at the bottom of the slide, the teacher will see the number of students who do not understand the content of this slide and give a more detailed explanation. As teachers and students are not in the same space, real-time interaction is difficult in previous online teaching mode. Teachers are unable to know whether the students understand the teaching content through students’ facial expressions and question feedback. Through the above-mentioned smart interactive functions, the students may ask the teacher for help in time. The qualitative research will be designed in the future to link each smart interactive tool to different type of interactions and the corresponding instructional strategies to analyze student satisfaction in depth.

After class, teachers can view the students’ interaction data, including the accuracy rate of class quiz answers, the students who posted the question barrage, and who did not understand a certain slide of PowerPoint. Meanwhile, some other data obtained from the smart interactive tools, such as the time taken by students to read the preview and review materials, and the accuracy rate of quiz answers, the teacher could evaluate whether the students complete the learning tasks well, whether they fully understand the teaching content of the course, etc. The teachers will communicate with the student who did not participate in the interaction or did not complete the preview and review tasks, through WeChat. After this kind of in-depth communication, students would know that the teacher is paying attention to them, so they will naturally keep attention to learning online courses. After the teachers communicated with several students who did not actively participate in interaction or did not complete the homework, the student’s enthusiasm in class and the completion of homework were improved. Through data analysis after class, the teachers would also in-depth communicate with those students whose learning ability is found to be poor and help them ameliorate their learning strategy and solve their problems.

Comparing to the student number and average score of different band scores, no one failed in the online course, but there were three academically lower-performing students who failed in the traditional course. The number and average score of online students in the band score from 60 to 69 were higher than those of traditional course. These results suggested that using the online smart interactive tools, the teachers can easily find those students with lower learning motivation, and provide personalized guidance in time, so that these students could finally pass the exam smoothly. However, traditional teaching cannot grasp the learning situation of each student, resulting in the students with learning difficulties who cannot be taken care of by the teacher in class could not pass the exam. In the band score above 70, no significant differences were observed in the average score and student number between the two teaching modes. The final exam average score of overall and of the academically lower and higher-performing students between the two teaching modes showed no significant differences as well, which indicated online teaching could achieve the same teaching effect as traditional face-to-face teaching by improving instructional design and increasing teacher-student interaction. To increase students’ enthusiasm for online learning, we evaluate the interactions in class *via* formative scores, including attendance rate, the accuracy rate of quiz answers, class participation, etc., and count the formative score in the final score. Since the final score of last year’s traditional course did not include the formative score, we could not compare this part. This study was implemented in an elective course, which might have a potential sampling bias. However, the comparisons were performed between the students from traditional and online course who voluntarily chose this course and have the similar motivation to follow this course. The above results showed the optimized online course with smart interactive tools could improve the learning effectiveness for the academically lower-performing students in this course. Furthermore, we will collect more evidence from other courses and conduct comparative analysis.

There are also some drawbacks to smart interactive tools. For example, the functions of Rain Classroom need to be further developed in the future, such as real-time audio or video interaction. The new version Rain classroom 4.4 updated in 2021 already allow teachers and students to access microphones and cameras at the same time with real-time online interactive functions so that there is no need to nest other interaction tools like Tencent Meeting simultaneously. In the spring semester of 2022, we plan to adopt a blended teaching mode of this course. Based on the face-to-face synchronous interaction, the smart interactive tools will be used online to enhance learner-content interaction and instructor-learner interaction, optimize the teaching strategy in time *via* the feedback from data analysis, and increase students’ learning motivation and effectiveness.

In summary, by using the optimized online teaching mode with smart interactive tools in higher education, the interaction can be perfectly realized, which ensures the online learning effectiveness of academically lower-performing students. Meanwhile, teachers can also know the learning status in which each student is and perform personalized guidance accordingly with the data analysis function of smart interactive tools to increase students’ learning motivation. The promotion of online teaching with smart interactive tools in higher education will help optimize the teaching ecosystem gradually, cultivate students’ self-study ability, realize personalized teaching, and improve the exam passing rate in the era of big data.

## Data Availability Statement

The original contributions presented in the study are included in the article/supplementary material, further inquiries can be directed to the corresponding author.

## Ethics Statement

Written informed consent was obtained from the individual(s) for the publication of any potentially identifiable images or data included in this article.

## Author Contributions

PW, TM, L-BL, and Y-XX designed and prepared the materials. PW, CS, and PA performed the data collection and analysis. PW wrote the first draft of the manuscript. All authors contributed to the article and approved the submitted version.

## Conflict of Interest

The authors declare that the research was conducted in the absence of any commercial or financial relationships that could be construed as a potential conflict of interest.

## Publisher’s Note

All claims expressed in this article are solely those of the authors and do not necessarily represent those of their affiliated organizations, or those of the publisher, the editors and the reviewers. Any product that may be evaluated in this article, or claim that may be made by its manufacturer, is not guaranteed or endorsed by the publisher.
